# Supplementary material for: Albumin-binding dendrimer-conjugated siRNA enables safe and effective gene silencing throughout the central nervous system
Source: Nucleic Acids Res. 2026 Sep 30;54(18):gkag926. doi: 10.1093/nar/gkag926 (PMC13624663; doi:10.1093/nar/gkag926)
Supplement: Supplementary file 1 [file gkag926_supplemental_file.pdf]

Supplementary Data for

Albumin-binding dendrimer-conjugated siRNA enables safe and effective gene silencing throughout the central nervous system

Supplementary Table S1. Detailed sequences and chemical patterns of oligonucleotides used in this study. Notations are as follows for modifications used: (#: PS backbone, m: 2'-omethyl, f: 2'-fluoro, C12: hexaethylene spacer, C6: triethylene spacer, SB: symmetrical branching, V: (E)-vinylphosphonate), "Unc" – unconjugated, "Di" – Divalent, "D" – Dendrimer.

Gene	Oligo ID	Sequence (5'-3')	Expected M.W.	Observed M.W.
HTT/Htt	HTT- Antisense	V(mU)#(fU)#(mA)(fA)(fU)(fC)(mU)(fC)(mU)(fU)(mU)(fA)(mC)(fU)#(mG)#(fA)#(mU)#(mA)#(mU)#(fA)#(mU)	6935.6	6935.6
	HTT- Sense-Unc	(dT)(dT)(mU)#(mC)#(mA)(fG)(mU)(fA)(mA)(fA)(mG)(fA)(mG)(mA)(mU)(fU)#(mA)#(mA)	5974.1	5973.8
	HTT- Sense-Di	(mU)#(mC)#(mA)(fG)(mU)(fA)(mA)(fA)(mG)(fA)(mG)(mA)(mU)(fU)#(mA)#(mA)(dT)(dT)-divalent	12340.2	12341.5
	HTT- Sense-D	(C12)(SB)(C6)(SB)(dT)(dT)(mU)#(mC)#(mA)(fG)(mU)(fA)(mA)(fA)(mG)(fA)(mG)(mA)(mU)(fU)#(mA)#(mA)	7852.1	7853.5
JAK1/Jak1	JAK1- Antisense	V(mU)#(fA)#(mU)(fC)(fG)(fC)(mU)(fU)(mG)(fU)(mA)(fG)(mC)(fU)#(mG)#(fA)#(mU)#(mG)#(mU)#(fC)#(mU)	7022.7	7021.9
	JAK1- Sense-Di	CyMN3#(mU)#(mC)#(mA)(fG)(mC)(fU)(mA)(fC)(mA)(fA)(mG)(mC)(mG)(fA)#(mU)#(mA)(dT)(dT)-Divalent	13534.9	13535.0
	JAK1- Sense-D	(C12)(SB)(C6)(SB)(dT)(dT)(Cy3)#(mU)#(mC)#(mA)(fG)(mC)(fU)(mA)(fC)(mA)(fA)(mG)(mC)(mG)(fA)#(mU)#(mA)	8326.0	8327.9
MECP2/Mecp2	MECP2- Antisense	V(mU)#(fA)#(mU)(fC)(fG)(fG)(mG)(fA)(mA)(fG)(mC)(fU)(mU)(fU)#(mG)#(fU)#(mC)#(mA)#(mG)#(fU)#(mU)	7084.8	7085.0
	MECP2- Sense-Di	(mA)#(mC)#(mA)(fA)(mA)(fG)(mC)(fU)(mU)(fC)(mC)(mC)(mG)(fA)#(mU)#(mA)(dT)(dT)-Divalent	12162.0	12163.2
	MECP2- Sense-D	(C12)(SB)(C6)(SB)(dT)(dT)((mA)#(mC)#(mA)(fA)(mA)(fG)(mC)(fU)(mU)(fC)(mC)(mC)(mG)(fA)#(mU)#(mA)	7763.0	7764.6
APP/App	APP- Antisense	V(mU)#(fG)#(mA)(fG)(fA)(fA)(mU)(fU)(mC)(fU)(mU)(fG)(mG)(fU)#(mA)#(fA)#(mU)#(mU)#(mG)#(fA)#(mU)	7094.8	7094.8
	APP- Sense-Unc	(mU)#(mU)#(mA)(fC)(mC)(fA)(mA)(fG)(mA)(fA)(mU)(mU)(mC)(fU)#(mC)#(mA)(dT)(dT)	5846.9	5846.9
	APP- Sense-Di	(mU)#(mU)#(mA)(fC)(mC)(fA)(mA)(fG)(mA)(fA)(mU)(mU)(mC)(fU)#(mC)#(mA)(dT)(dT)-Divalent	12085.9	12087.1
	APP- Sense-D	(C12)(SB)(C6)(SB)(dT)(dT)(mU)#(mU)#(mA)(fC)(mC)(fA)(mA)(fG)(mA)(fA)(mU)(mU)(mC)(fU)#(mC)#(mA)	7724.9	7727.5
NTC	NTC Antisense	V(mU)#(fA)#(mA)(fU)(fC)(fG)(mU)(fA)(mU)(fU)(mU)(fG)(mU)(fC)#(mA)#(fA)#(mU)#(mC)#(mA)#(fU)#(mU)	6974.7	6974.7
	NTC Sense – D	(C12)(SB)(C6)(SB)(dT)(dT)(mU)#(mU)#(mG)(fA)(mC)(fA)(mA)(fA)(mU)(fA)(mC)(mG)(mA)(fU)#(mU)#(mA)	7789.0	7792.5
	NTC Sense – Unc	(mU)#(mU)#(mG)(mA)(mC)(fA)(fA)(fA)(mU)(fA)(mC)(mG)(mA)(mU)#(mU)#(mA)(dT)(dT)	6974.7	n.a.
HTT/Htt Cy3	HTT- Sense-Unc	(dT)(dT)(Cy3)#(mU)#(mU)#(mG)(fA)(mC)(fA)(mA)(fA)(mU)(fA)(mC)(mG)(mA)(fU)#(mU)#(mA)	6434.3	6434.6
	HTT- Sense-Di	CyMN3#(mU)#(mC)#(mA)(fG)(mU)(fA)(mA)(fA)(mG)(fA)(mG)(mA)(mU)(fU)#(mA)#(mA)-DIO	12416.2	12415.6
	HTT- Sense-D	(C12)(SB)(C6)(SB)(dT)(dT)(Cy3)#(mU)#(mC)#(mA)(fG)(mU)(fA)(mA)(fA)(mG)(fA)(mG)(mA)(mU)(fU)#(mA)#(mA)	8375.4	8377.3

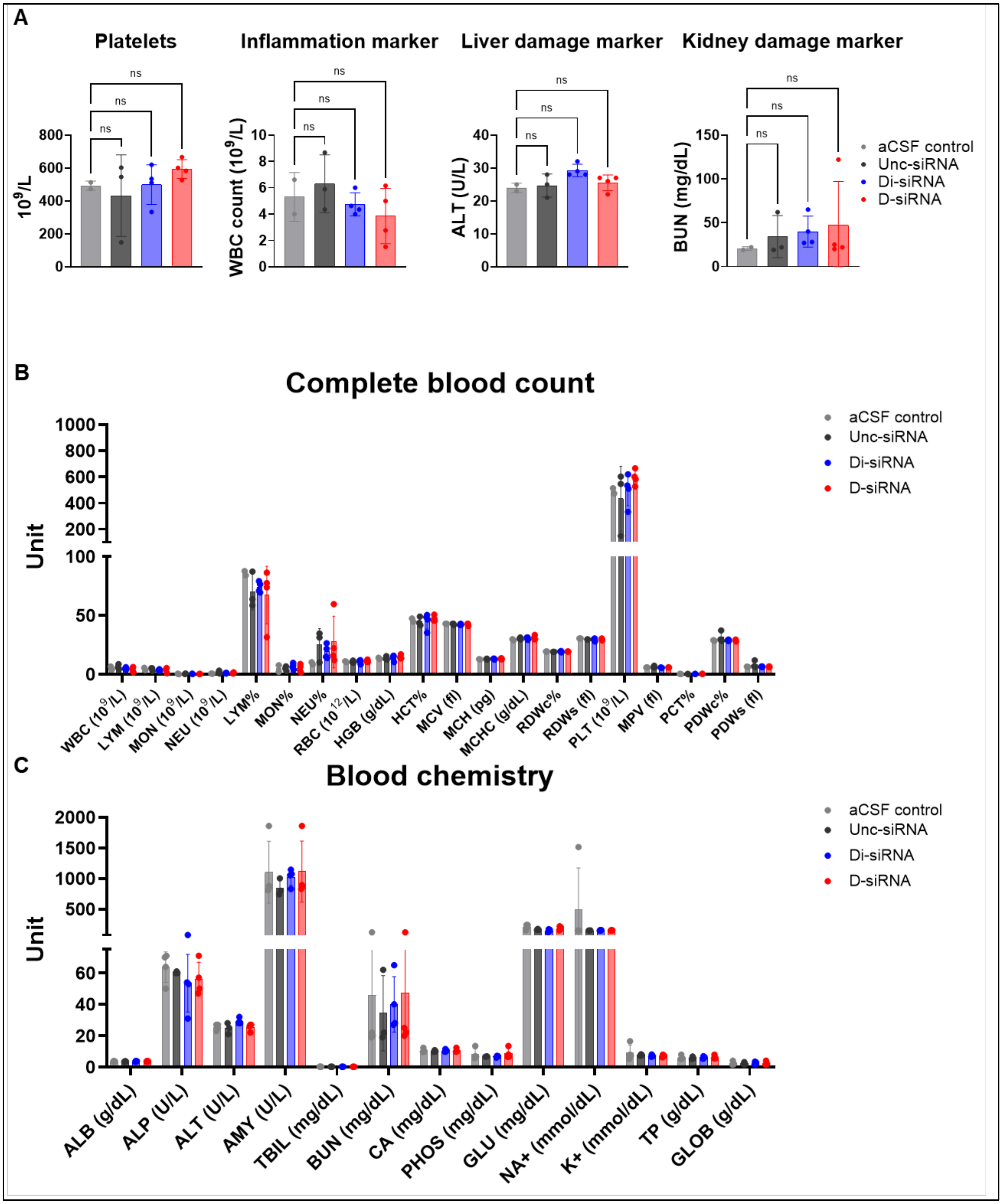

Supplementary Figure S1. Complete blood count (CBC) and blood chemistry readouts show a favorable safety profile for D-siRNA following CSF administration. (A) Representative markers from CBC and blood chemistry at 24h post ICV injection, showing no significant changes between the various siRNA chemistries: Platelet count, white blood cell count as marker of inflammation, ALT as marker of liver damage, and BUN as a measure of kidney damage. Full data sets of **(B)** CBC and **(C)** blood chemistry readouts. Data represented as mean ± S.D.; n=2-3 mice per group; Statistical analysis was performed in GraphPad Prism: one-way ANOVA analysis of all groups against aCSF control group (ns = non-significant, * p<0.05, ** p<0.01, *** p<0.001, **** p<0.0001).

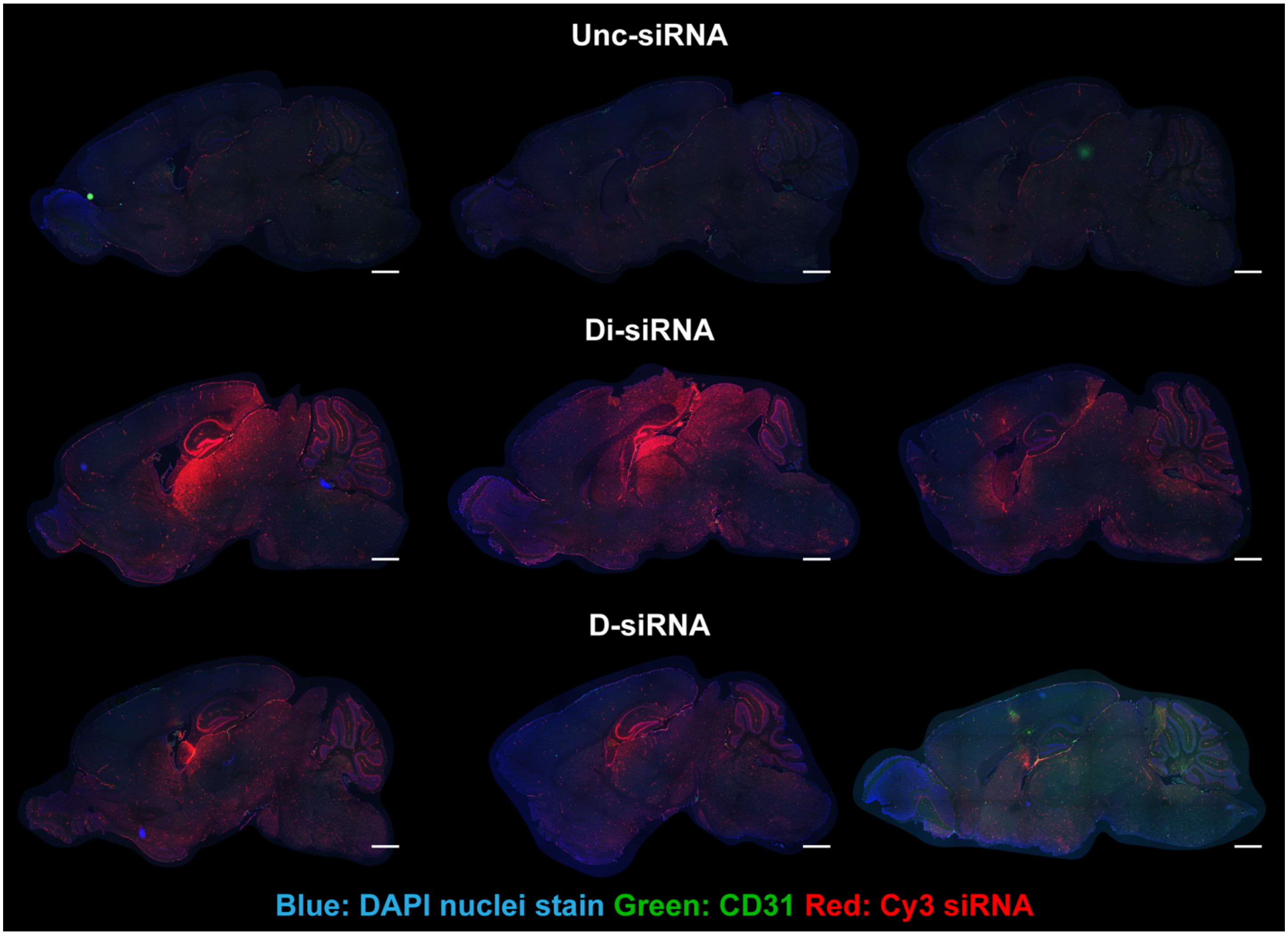

Supplementary Figure S2. Individual brain images corresponding to Figure 2. Scale bars: 1 mm.

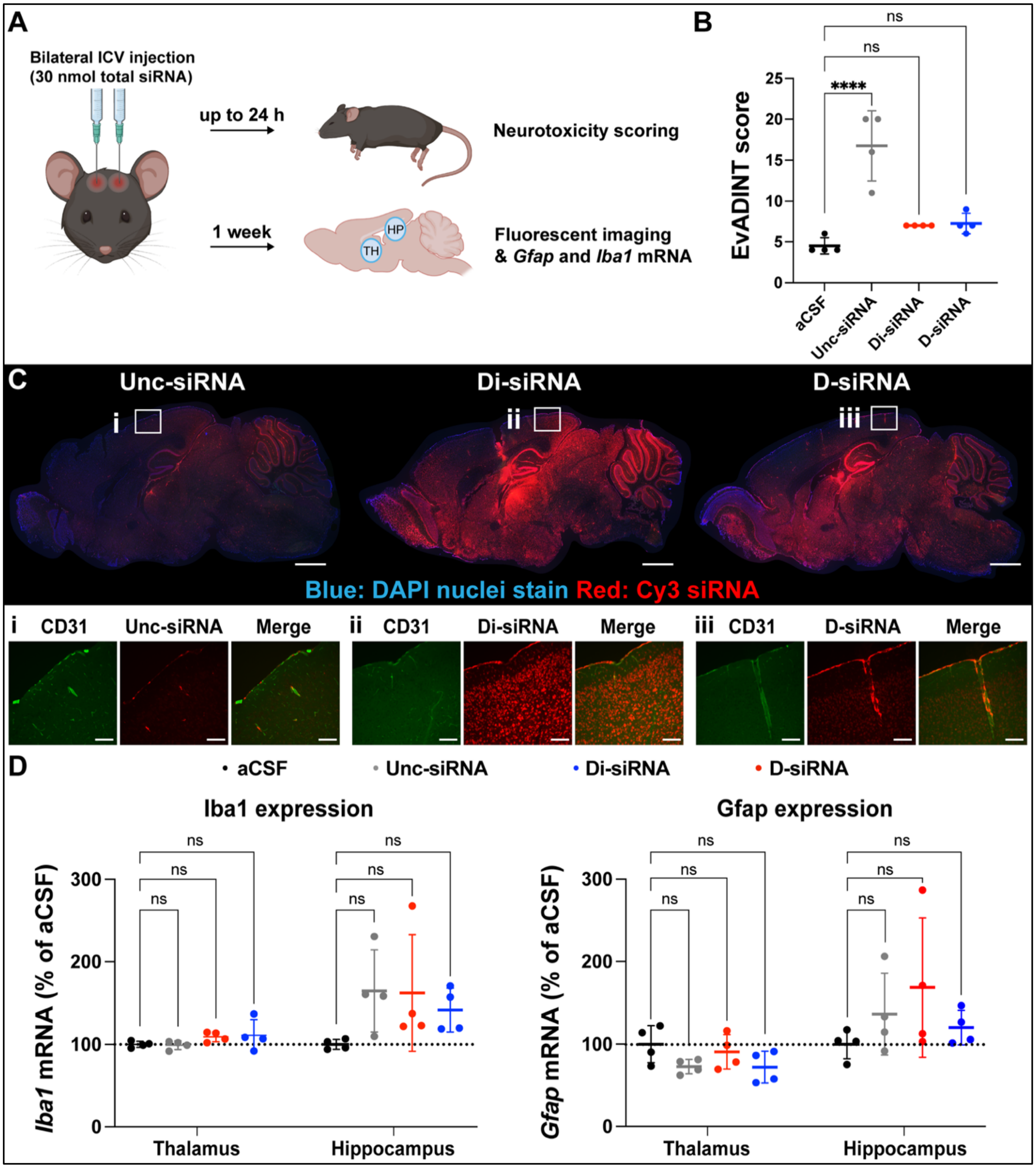

Supplementary Figure S3. Safety and imaging evaluation one week after high-dose ICV administration of siRNA scaffolds in mice. **(A)** Experimental schematic for the study of intracerebroventricular injection of a 30 nmol dose of Cy3-labeled siRNAs; acute neurotoxicity was assessed for up to 24 h after ICV injection, and tissue distribution and neuroinflammation markers were evaluated one week after injection. **(B)** EvADINT scoring system. Higher scores indicate higher neurotoxicity. **(C)** **(top)** Representative fluorescence imaging of brain tissue one week post-ICV injection of Cy3-labeled Unc-, Di-, or D-siRNAs (red=Cy3 siRNA, blue= DAPI staining). Scale bars: 1 mm. **(bottom)** Colocalization of siRNAs with vascular marker CD31 (green). Scale bars: 100 μm. **(D)** Levels of neurotoxicity mRNA markers in the thalamus and hippocampus. Data represented as mean ± S.D.; n=4 mice per group; Statistical analysis was performed in GraphPad Prism: one-way ANOVA analysis of all groups against aCSF control group (ns = non-significant, **** p<0.0001); dotted line on **(D)** represents 100% remaining mRNA expression.

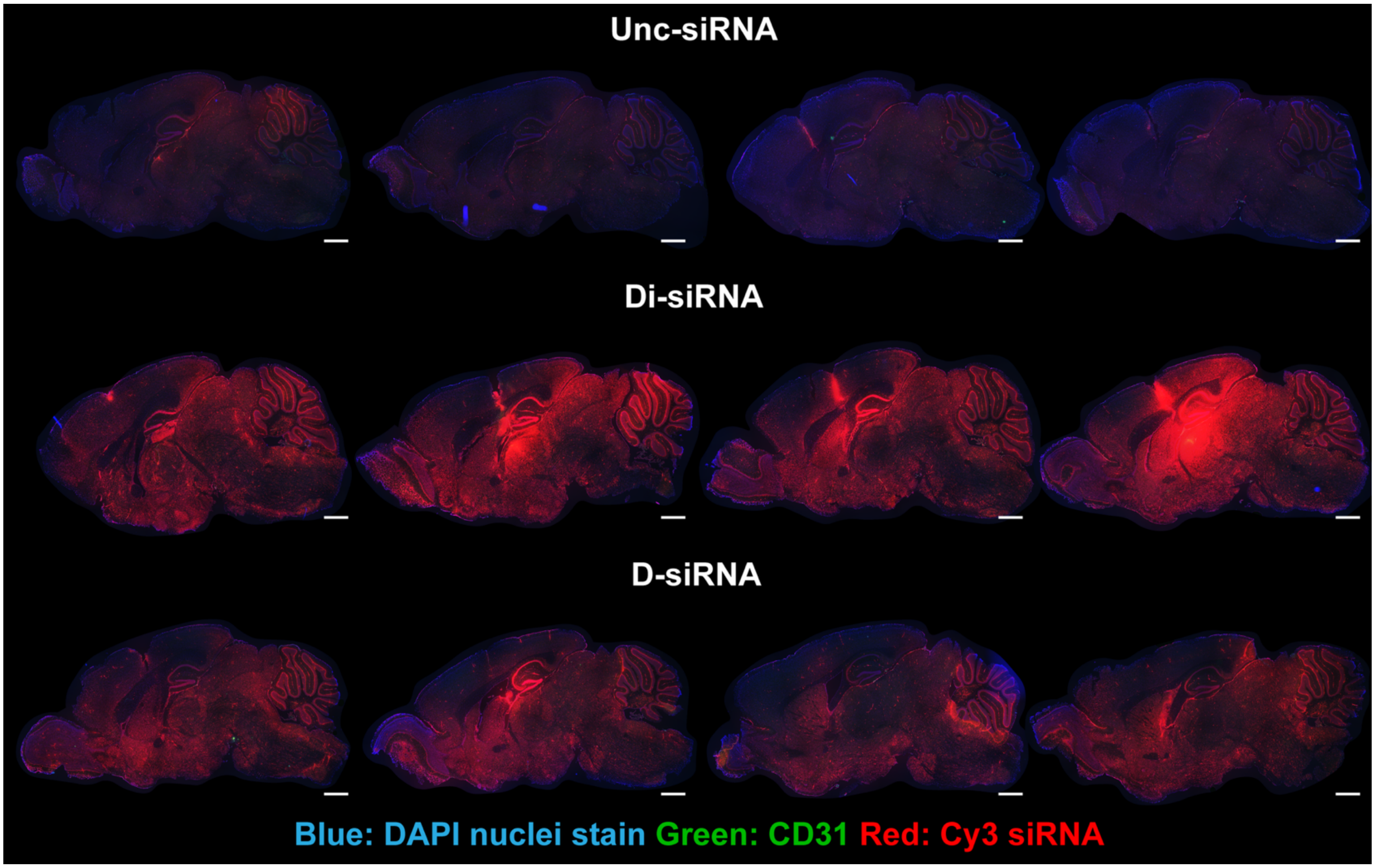

Supplementary Figure S4. Individual brain images corresponding to Supplementary Figure S3. Scale bars: 1 mm.

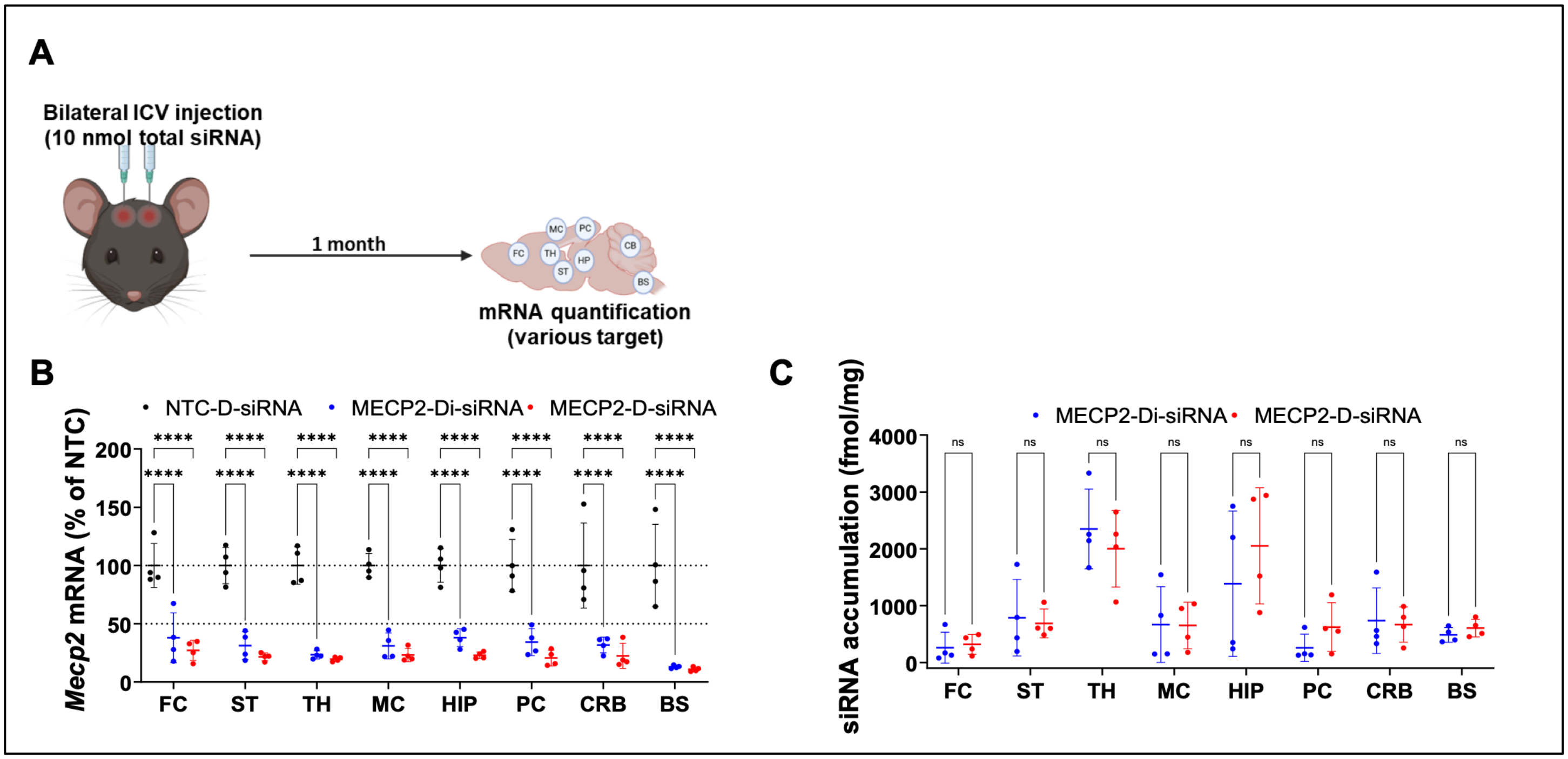

Supplementary Figure S5. D-siRNA achieves similar efficacy to Di-siRNA targeting *Mecp2* at one-month post ICV injection. (A) Schematic of *Mecp2*-targeting siRNA efficacy study following bilateral ICV injection of 10 nmol dose at one-month post injection (equivalent to 130 µg of siRNA). **(B)** mRNA levels of *Mecp2* compared to NTC-D control and **(C)** siRNA accumulation levels comparing D-siRNA and Di-siRNA. All study groups are n=4 mice/group. Statistical analysis was performed in GraphPad Prism: two-way ANOVA analysis followed by Dunnett's multiple comparisons of all groups against NTC control group for **(B),** and followed by Bonferroni's multiple comparisons between Di- and D-siRNA groups for **(C)** (ns = non-significant, * p<0.05, ** p<0.01, *** p<0.001, **** p<0.0001). (Data represented as mean ± S.D.; mRNA measured by the Quantigene 2.0 Assay; tissue accumulation measured by PNA assay; dotted line on **(B)** represents 100% and 50% remaining mRNA expression; graphical elements created with BioRender).

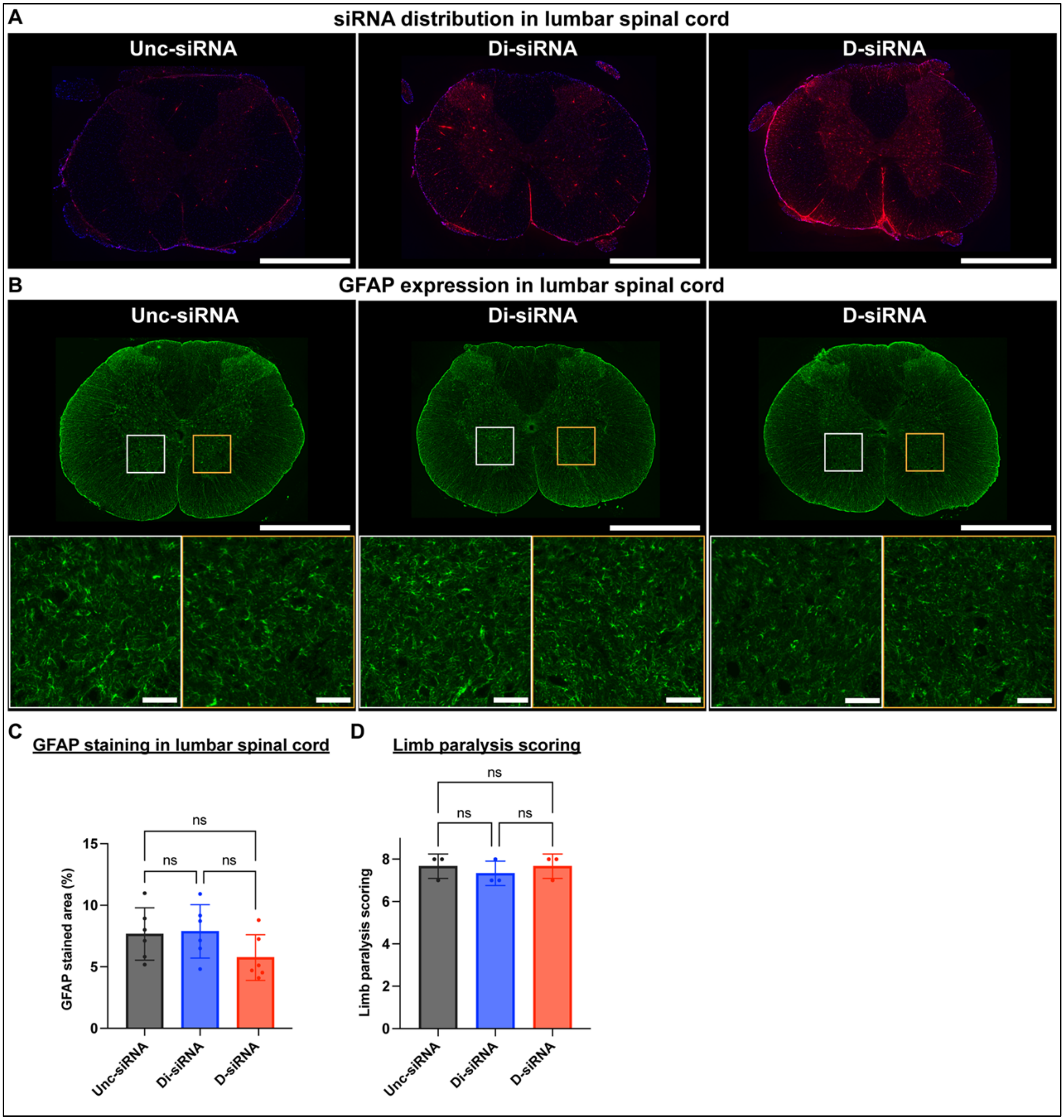

Supplementary Figure S6. Distribution of siRNA and assessment of astrogliosis in the lumbar spinal cord. (A) Representative images of the siRNA distribution in the lumbar spinal cord 48 hours after IT injection. **(B)** Representative images of GFAP expression in the lumbar spinal cord 48 hours after IT injection. **(C)** The comparison of GFAP staining area in the lumbar spinal cord. **(D)** The comparison of the limb paralysis scoring 24 hours after IT injection. (Data represented as mean ± S.D.; n=3 rats per group; statistical analysis was performed in GraphPad Prism: one-way ANOVA analysis followed by Tukey's multiple comparisons test across the groups (ns = non-significant); GFAP quantification by immunostaining; scale bars represent 1 mm in **(A)** and the top panel of **(B)** and 100 µm in the bottom panel of **(B)**; limb paralysis scoring was blinded)

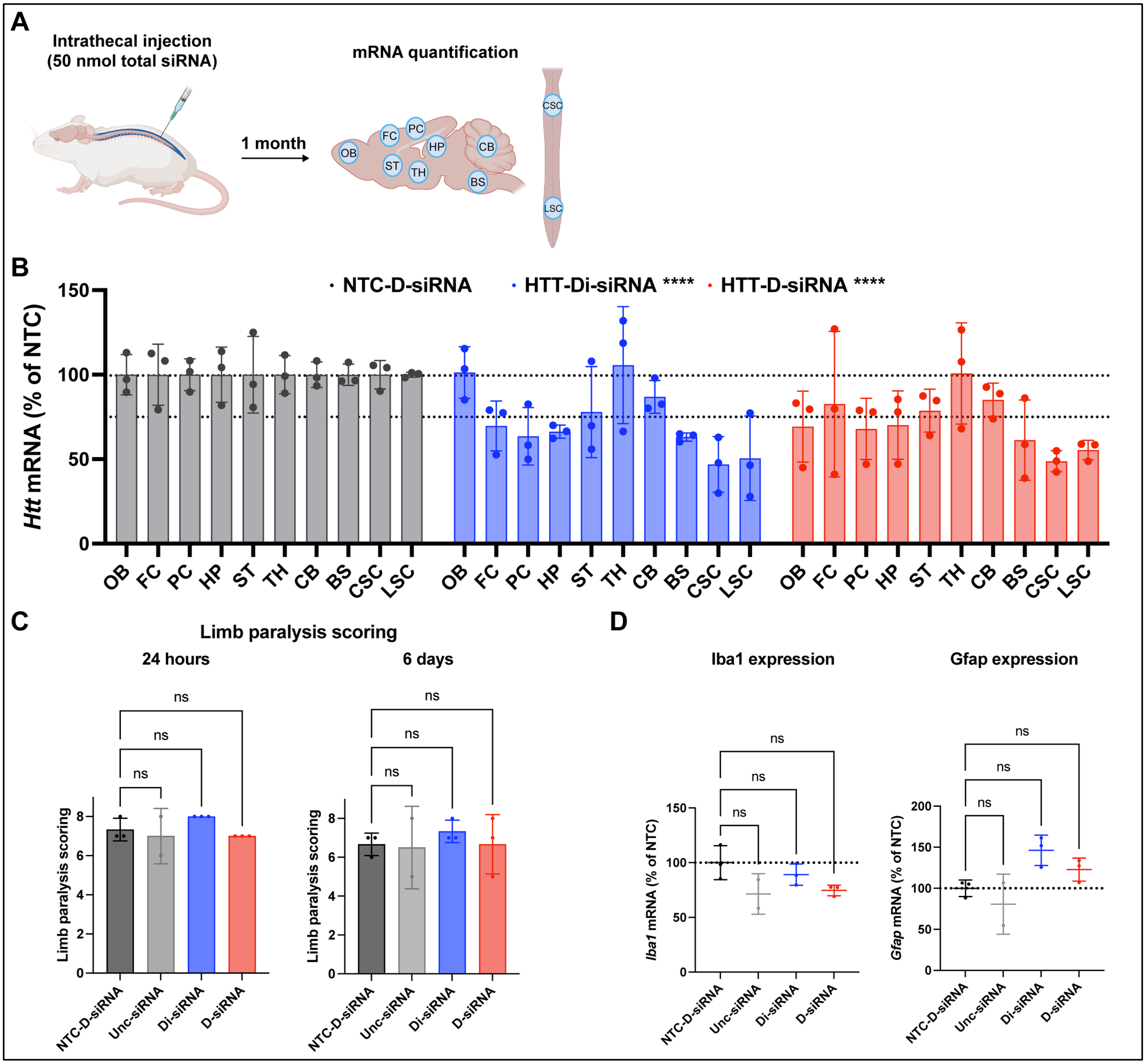

Supplementary Figure S7. D-siRNA shows comparable silencing of *Htt* mRNA to Di-siRNA
(A) Experimental schematic for the study of intrathecal injection of 50 nmol dose of *Htt*-targeting Cy3-siRNAs; mRNA levels were assessed 1 month after IT injection of Di- and D-siRNA. **(B)** Silencing data of *Htt* mRNA in various CNS regions. **(C)** Limb paralysis scores at 24 hours and 6 days post-injection. **(D)** Levels of neurotoxicity mRNA markers in the lumbar spinal cord (*Iba1* for microgliosis and *Gfap* for astrogliosis). All study groups are n=2-3 rats/group. Statistical analysis was performed in GraphPad Prism: two-way ANOVA analysis of all groups against NTC control group for **(B)** and one-way ANOVA analysis of all groups against NTC control group for **(C)** and **(D)** (ns = non-significant, **** $p<0.0001$). (Data represented as mean ± S.D.; mRNA measured by the Quantigene 2.0 Assay; dotted line on **(B)** represents 100% and 75% remaining mRNA expression; graphical elements created with Biorender).

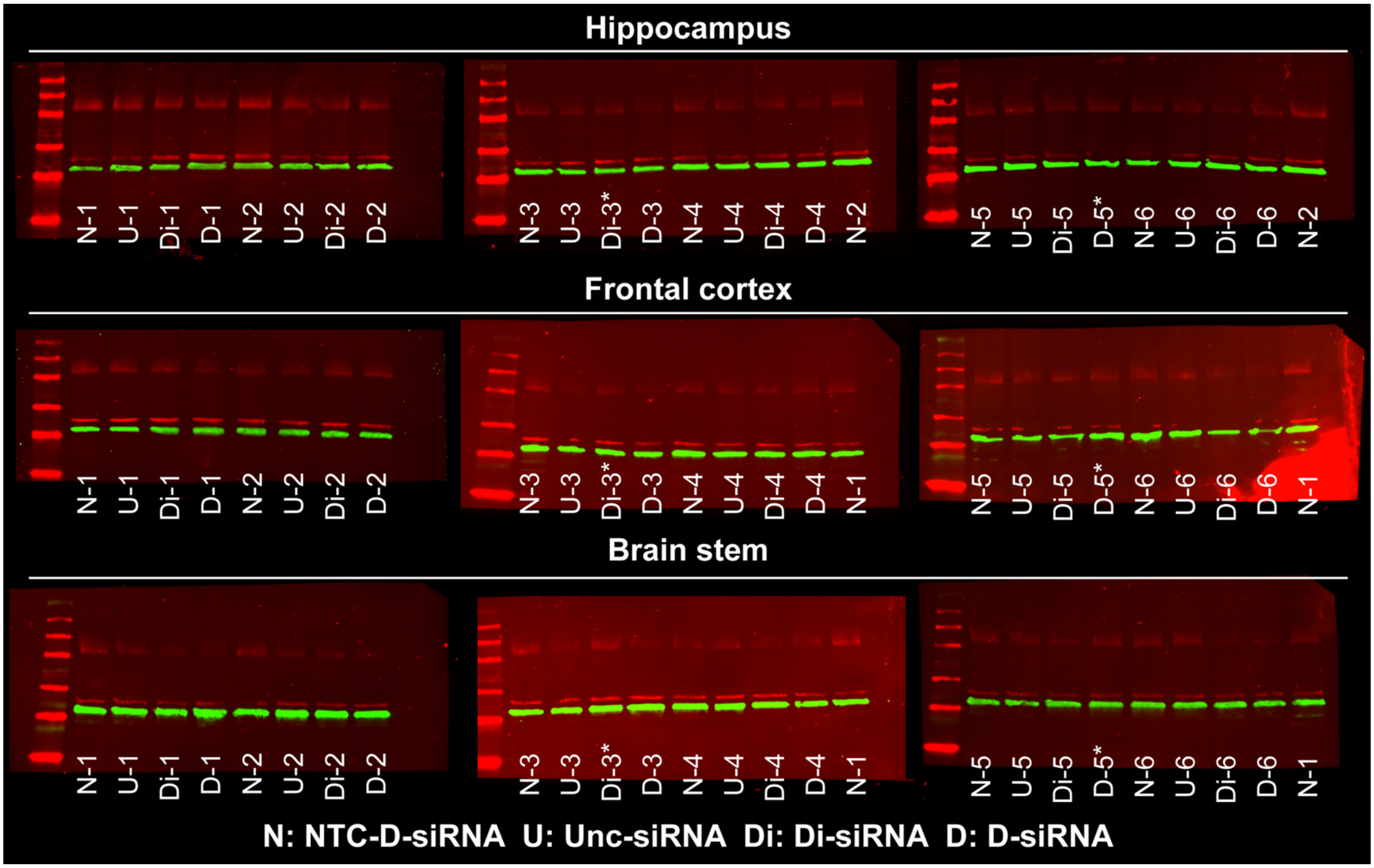

Supplementary Figure S8. Original western blot data for Figure 5C. Asterisks indicate misinjections; these animals were excluded from the analysis.
